# Immature Observations

**Published:** 1868-06

**Authors:** Geo. B. Willson

**Affiliations:** Port Huron, Mich.


### Immature Observations.

BY GEO. B. WILLSON,* M. D., PORT HURON, MICH.

There is incalculable injury done the profession every little while whenever a new remedy is introduced, by a host of superficial observers and ready writers, who rush into print through the medical journals to give their evidence in favor of the efficacy of the new medicine in every conceivable form of disease or injury. In many cases—nay, I might say in a majority—the writers have only tried the remedy once or twice, and then, instead of waiting to see if the cure be permanent or not, they publish a report of it, perhaps the same day; very often, at all events, while the patient is yet in the convalescent state and before recovery is complete. The report of cases in such a manner, or even very soon after what is regarded as complete recovery, is highly reprehensible. These persons seem fearful that some one else may have had a similar case and will publish it before they do. But it is with regard to new remedies, or the trial of old remedies in some new way, that the greatest nuisance arises. It will be recollected how, on the introduction of glycerine into general use a few years ago, the journals were crammed to overflowing with accounts of its wonderful effects and the variety of its applications. From every part of the country, and in every medical journal, and in every number of every journal for about a year, there came accounts of the uses made of glycerine, and of its efficacy in diseases when applied externally or exhibited internally. It is no exaggeration to say that it was recommended in every disease ever heard of as prevailing in this country. As an external application in skin diseases of every kind there came reports of its trial with successful results. Its soothing and anodyne properties as an eye wash devoid of all irritation were duly vouched for; and its efficacy, in short, in every disease, not excepting consumption, in which some one found it an excellent substitute for cod liver oil. Any one giving credence to one-half of what was written about it could come to no other conclusion than that nearly or quite one-half of the materia medica could now be dispensed with and glycerine substituted. Admit all this flood of positive evidence not one word of negative found its way, nor

* Deceased.

even to this day, though hundreds must have seen the fallacy of most of the statements, has there been anything of the negative kind published. Now where such a course is pursued about a remedy, and every new remedy goes through the same round that glycerine has, how is one to know from journals or books what the remedy is good for at all? To say that a remedy is equally good for everything, is to say that it is really good for nothing. Most of those letters and notices concerning its varied powers were written after only one successful case. At the present time, and then also, a chapter of negative evidence on the effects of glycerine and kindred articles was much more needed than that of a positive kind. To such an extent has this mistake been carried, that the same is true of almost every article of the materia medica. The most valuable paper that could be published on any one of them would be an enumeration of what it would *not* do.

The very valuable medicine perchloride of iron had to run the gauntlet like glycerine, and came near being swamped with the flood of evidence in its favor. Both of these articles now require a long chapter of negative evidence to restore them to their proper place and estimation. But in fact every article of the materia medica needs the same. As an instance of the confusion to which so much positive evidence leads, I give my own experience:—A few years ago I was appointed by our State Society chairman of a committee to report on *the action of quinine.* I set about my task by attempting to collect and classify what had been written on the subject, but I soon found that impossible. I became satisfied that I could produce good authority (members of the profession in good standing) proving it to possess almost every power attributed to each of the common classes of medicine, and also proving it to be a specific in every disease of the nosology, and in every stage of each disease. There was little or no conflicting evidence to be found—all the evidence was of the positive kind. There were one or two writers who ventured a negative as to its exhibition in large doses in the second stage of typhoid and continued fevers, but their opinions met with much opposition from other observers. Hence quinine had all powers and virtues, and cured all diseases, and was admissible and desirable in every stage and every condition of every disease. This was the inevitable conclusion derived from consulting the written authorities—text books, monographs, and medical periodicals. What was I to do under such circumstances? To give the amount of our knowledge on the action and virtues of quinine as derived from positive evidence would require a volume. Acknowledging my inability therefore to do justice to the subject, I began to write down what I thought quinine would *not* do. The chapter of positive evidence was full; it would do everything. The chapter of negative evidence was a blank, so I commenced the filling of it. I could get no assistance in that work; I had merely to write from my own experience and observation. But my observations were so limited that I did not deem them worthy of presentation.

Let any one take any other valuable remedy and see if he does not meet with the same difficulty that I did with quinine. We really know less of the therapeutic powers of our valuable medicines now than we did ten years ago—and why? Because what we knew then, and what we have learned since, is so overrun with unreliable statements that we find it impossible to separate the true from the false. About two years ago a physician reported in a journal that he found lupulin to be a specific for *delirium tremens*. He gave it in large doses, having in one instance given as high as six pounds! The enormous dose caused some parties to make inquiry for further information, which came in the next issue of the journal, to the effect, that not the lupulin itself, but a tincture made with six ounces of lupulin, and six pints of brandy, had been given!

I also saw it stated in one or two journals that gonorrhœa could be cured effectually in forty-eight hours by extract of conium, administered in twelve grain doses every two hours. Notwithstanding the enormous doses, the editors never expressed suspicion or gave a hint of caution. And to this day I have not seen a contradiction or explanation of the statement.

Again, wonderful cures of every disease were effected all over the country by tincture of cannabis Indica during the first few months after its introduction. Where are they now? Those who reported most of them could not tell the effects of the medicine from the workings of nature, and so became discoverers, and must needs astonish the world forthwith by publishing their discoveries.

I could publish a long list of negative evidence with regard to cannabis Indica, but it is unnecessary now as the article seems to be sinking into obscurity. Of perchloride of iron, too, a long list of negative evidence is wanted. The article is a valuable one, and ought to be disencumbered from the trash that correspondents have heaped upon it.—*Boston Medical and Surgical Journal.*

